# Supplementary material for: An exploratory study of patients’ experiences with and reasons for using one virtual-only telecontraceptive platform in the United States in 2020–2021
Source: Reprod Health. 2025 Dec 3;22:245. doi: 10.1186/s12978-025-02181-0 (PMC12676766; doi:10.1186/s12978-025-02181-0)
Supplement: Supplementary file 1 — Supplementary Material 1 [file 12978_2025_2181_MOESM1_ESM.docx]

Appendix I Survey Instrument

**Experiences getting birth control from [the platform] during the COVID-19 pandemic**

*Inclusion criteria*

- How old are you?
- [dropdown whole number 18-49]
- When did you **first** use [the platform] for birth control? If you’re not certain, please select the approximate month and year.
- [list month]
- [list year]

*[INFORMED CONSENT]*

*Patient History and Characteristics*

*The first set of questions will help us understand more about people who get their birth control from [the platform]. Your responses in this survey are confidential.*

- How do you identify your race and/or ethnicity? Please select all that apply.
  - American Indian or Alaskan Native
  - Asian
  - Black or African-American
  - Hispanic, Latina, Latinx, or Spanish origin
  - Pacific Islander
  - White
  - Another race, ethnicity, or origin (please specify) *[textbox]*
  - Prefer not to answer
- In what state do you currently live?
  - [list 50 states + DC]
  - Prefer not to answer
- Is this the same state you lived in when you used [the platform] for birth control services **for the first time**?
  - Yes
  - No
  - Prefer not to answer

*[If no]* In what state did you live when you **first** used [the platform] for birth control services?

- [list 50 states + DC]
- Prefer not to answer
- What best describes your current gender identity? (Select all that apply)
  - Agender
  - Cisgender woman (a person that identifies as a woman and was assigned female sex at birth)
  - Genderqueer
  - Man
  - Non-binary
  - Transgender Man
  - Transgender Woman
  - Two-Spirit (feel free to include your tribe’s specific language for your identity, if you would like) *[textbox]*
  - Woman
  - Additional gender category, please specify: *[textbox]*
  - Prefer not to answer
- What is your current relationship status?
  - Never married, single
  - Never married, in a relationship and not living with partner
  - Never married, in a relationship and living with partner
  - Married
  - Widowed
  - Separated
  - Divorced
  - Other (please describe)
- How many people live in your household (including you)?
  - (Drop down number 1-10+)
- What is your total annual household income?
  - Please write your income as a whole number **with no dollar sign (e.g., 3,000 or 24,000 or 400,000): *[textbox]***
  - I don’t know
  - Prefer not to answer
- Do you currently have health insurance or are you covered by someone else’s insurance?
- Yes
- No
- Don’t know
- Prefer not to answer

*[If yes]* What type of health coverage? (Select all that apply)

- Private health insurance, including insurance that you have through your or your partner’s job, your school or college, your parents
- Military insurance (TRICARE or VA health care)
- Indian Health Service
- Insurance through Obamacare (Affordable Care Act, ACA)
- Medicaid, Medicare, Medi-Cal, Title XIX, or any other state or federal sponsored health plan for low-income families
- Any other type of medical coverage or health insurance (please specify) [textbox]
- What is the highest grade in school that you have completed?
- No formal education
- Less than high school
- High school degree or GED
- Some college
- College degree
- Some professional school or advanced education
- Professional or advanced degree
- Prefer not to answer
- How would you describe your employment and/or student status now? (Select all that apply)
- Working part time
- Working full time
- Student (full time)
- Student (part time)
- Unemployed
- Retired
- Permanently disabled
- Taking care of home or family
- Other (please specify*) [textbox]*
- Prefer not to answer
- Which birth control method(s), if any, are you **currently** using? (Select all that apply)
- None—I am not using any birth control method
- Withdrawal (pulling out)
- Birth control pills
- Vaginal ring (NuvaRing, Annovera)
- Shots or injections (Depo-Provera or “Depo”)
- Patch (OrthoEvra patch, Xulane)
- Hormonal IUD (Mirena, Skyla, Liletta, Kyleena)
- Copper IUD (Paragard)
- Implant (Implanon/Nexplanon)
- Male condoms (“external condoms”)
- Female condoms (FC2, “internal condoms”)
- Vaginal barrier methods (diaphragm, sponge)
- Spermicide (cream, gel, film, foam, suppositories)
- Fertility awareness methods (rhythm method, cycle beads, periodic abstinence)
- Tubal ligation (sterilization)
- Partner's vasectomy
- Emergency contraception (also called “EC”, “morning after” pill, Plan B, Ella, Next Choice, EContra, EZ, My Way, After Pill)
- Another method (please tell us) *[textbox]*
- Prefer not to answer
- *[If not “none” or “prefer not to answer” “Withdrawl” “Fertility Awareness Methods” “Tubal ligation” “partner’s vasectomy” or “Another method”] [for each method selected above]* Where did you get your [current method] the last time you got it?
  - [the platform]
  - Another online telemedicine service (such as [Example 1], [Example 2], [Example 3], [Example 4], etc)
  - A doctor/clinic, after a **telemedicine** visit
  - A doctor/clinic, after an **in-person** visit
  - A doctor/clinic, **without any visit** (because it was a refill)
  - I received a prescription from a pharmacist
  - I bought it at a store (no prescription required)
  - Other (please tell us) *[textbox]*
  - Prefer not to answer
- **Before using [the platform]** for birth control, which birth control methods (if any) had you **ever** used, either to prevent pregnancy or for some other reason (please select all that apply)
  - None—I had not used any birth control before getting a method from [the platform]
  - Withdrawal (pulling out)
  - Birth control pills
  - Vaginal ring (NuvaRing, Annovera)
  - Shots or injections (Depo-Provera or “Depo”)
  - Patch (OrthoEvra patch, Xulane)
  - Hormonal IUD (Mirena, Skyla, Liletta, Kyleena)
  - Copper IUD (Paragard)
  - Implant (Implanon/Nexplanon)
  - Male condoms or external condoms
  - Female condoms, FC2, internal condoms
  - Vaginal barrier methods (diaphragm, sponge)
  - Spermicide (cream, gel, film, foam, suppositories)
  - Fertility awareness methods (rhythm method, cycle beads, periodic abstinence)
  - Tubal ligation (sterilization)
  - Partner's vasectomy
  - Emergency contraception (also called “EC”, “morning after” pill, Plan B, Ella, Next Choice, EContra, EZ, My Way, After Pill)
  - Another method (please tell us) *[textbox]*
  - Prefer not to answer
- *[If NOT none]* **In the month before getting a birth control method from [the platform] for the first time**, which birth control method(s) were you using, if any? Please select all that apply.
  - None—I was not using any birth control in the month before first getting a method from [the platform]
  - Withdrawal (pulling out)
  - Birth control pills
  - Vaginal ring (NuvaRing, Annovera)
  - Shots or injections (Depo-Provera or “Depo”)
  - Patch (OrthoEvra patch, Xulane)
  - Hormonal IUD (Mirena, Skyla, Liletta, Kyleena)
  - Copper IUD (Paragard)
  - Implant (Implanon/Nexplanon)
  - Male condoms or external condoms
  - Female condoms, FC2, internal condoms
  - Vaginal barrier methods (diaphragm, sponge)
  - Spermicide (cream, gel, film, foam, suppositories)
  - Fertility awareness methods (rhythm method, cycle beads, periodic abstinence)
  - Tubal ligation (sterilization)
  - Partner's vasectomy
  - Emergency contraception (also called “EC”, “morning after” pill, Plan B, Ella, Next Choice, EContra, EZ, My Way, After Pill)
  - Another method (please tell us) *[textbox]*
  - Prefer not to answer
- *[If above response is NOT “none”, “prefer not to answer”, “withdrawal, fertility awareness, partner’s vasectomy, tubal ligation, or another method] [for each method selected above]* **In the month before getting a birth control method from [the platform]** **for the first time**, where did you get your [recent method]?

Another online telemedicine service (such as [Example 1], [Example 2], [Example 3], [Example 4], etc)

- - A doctor/clinic, after a **telemedicine** visit
  - A doctor/clinic, after an **in-person** visit
  - A doctor/clinic, **without any visit** (because it was a refill)
  - I received a prescription from a pharmacist
  - I bought it at a store (no prescription required)
  - Other (please tell us)
  - Prefer not to answer
- Is there a method of birth control that you would like to use but you are not currently using?
  - Yes
  - No
  - Not sure
  - Prefer not to answer

[*If yes*] Which method(s) would you like to use but you are not currently using? Select all that apply.

- Withdrawal (pulling out)
- Birth control pills
- Vaginal ring (NuvaRing, Annovera)
- Shots or injections (Depo-Provera or “Depo”)
- Patch (OrthoEvra patch, Xulane)
- Hormonal IUD (Mirena, Skyla, Liletta, Kyleena)
- Copper IUD (Paragard)
- Implant (Implanon/Nexplanon)
- Male condoms (“external condoms”)
- Female condoms (FC2, “internal condoms”)
- Vaginal barrier methods (diaphragm, sponge)
- Fertility awareness methods (rhythm method, cycle beads, periodic abstinence)
- Tubal ligation (sterilization)
- Partner's vasectomy
- Emergency contraception (also called “EC”, “morning after” pill, Plan B, Ella, Next Choice, EContra, EZ, May Way, After Pill)
- Another method (please tell us) *[textbox]*
- Prefer not to answer

The next set of questions are about your experience using [the platform] **for the very first time for birth control.**

- Was [the platform] the **first** telemedicine service you have used (to get health care at a distance using technology, like a phone or computer) **for any health care need**?
  - Yes
- No
- Not sure
- Prefer not to answer

[*If no*] What other types of telemedicine services (receiving care at a distance using technology, like a phone or computer) have you used in the past (for any reason)? Please select all that apply.

- [the platform] (for something other than birth control)
- Another online telemedicine company (such as [Example 1], [Example 2], [Example 3], [Example 4], etc)
- I had a telemedicine visit with my regular doctor or clinic
- I had a telemedicine visit with a pharmacist
- Other (please specify) *[textbox]*
- When you got your birth control from [the platform], was this your **first time** using a telemedicine service (receiving care at a distance using technology, like a phone or computer) for **birth control** (counseling or method)?
  - Yes
  - No
  - Not sure
  - Prefer not to answer

*[If no]* What other types of telemedicine services (receiving care at a distance using technology, like a phone or computer) have you used before **for birth control**? Please select all that apply.

- Another online telemedicine service (such as [Example 1], [Example 2], [Example 3], [Example 4], etc)
- I had a telehealth visit with my regular doctor or clinic
- I had a telehealth visit with a pharmacist
- Other (please specify) *[textbox]*
- Prefer not to answer
- Did the COVID-19 pandemic play a role at all in choosing to use [the platform] for birth control?
  - Yes
  - No
  - Not sure
  - Prefer not to answer

*[If yes]* Please tell us more about how the pandemic played a role in your decision to use [the platform] for birth control. *[textbox]*

*[If unsure]* Please tell us more about why you are unsure about the role the pandemic played in choosing to use [the platform] for birth control. *[textbox]*

- How did you hear about [the platform]? Please select all that apply.
  - Advertisement
  - Article/news story
  - Website (please tell us: ____)
  - Friend
  - Family member
  - Other (please tell us:_____)
  - Prefer not to answer
- What were your reason(s) for choosing [the platform] for birth control services for the first time? Please select all that apply.
  - Convenience/easier to get birth control
  - To save time to not have to visit a clinic
  - To save money to not have to pay for a visit to a clinic
  - Doctor or clinic office hours were not convenient
  - It was hard to get time off from work, school, or to get childcare
  - It was hard to get a clinic or pharmacy
  - I didn’t have insurance
  - I didn’t want to use insurance
  - I didn’t have a regular doctor or clinic
  - To avoid going to an in-person clinic visit during the pandemic
  - Privacy or to get birth control without others knowing
  - I didn’t want to get a physical or pelvic exam in order to get birth control
  - Some other reason (please tell us)
  - Prefer not to answer

The **first time** you used [the platform] for birth control services, how sure were you about **which method** you wanted going into your visit?

- Very sure
- Somewhat sure
- Somewhat unsure
- Very unsure
- I don’t remember
- Prefer not to answer
- During your **first time** using [the platform] for birth control services, did you receive birth control **counseling**, where information was shared with you about different methods?
  - Yes
  - No
  - I don’t remember
  - Prefer not to answer
- During your**first time**using [the platform] for birth control services, did you have a video visit?
  - Yes
  - No
  - I don’t remember
  - Prefer not to answer

*[If yes*] How did the video visit with [the platform] compare to any past in-person visits for birth control that you have had?

- The video visit was better
- The video visit was the same
- The video visit was worse
- I’ve never had an in-person visit for birth control
- Prefer not to answer

*[If NOT prefer not to answer/never had a visit]* Please tell us why you thought the video visit was [better/worse/same] compared to an in-person visit. [*textbox]*

- During your**first time**using [the platform] for birth control services, what birth control method(s) did you **request**, if any? Please select all that apply.
  - Birth control pill
  - Patch
  - Ring
  - Emergency contraception
  - Other (please specify) [*textbox]*
  - I did not request a specific method
  - Prefer not to answer
- Were you seeking a new prescription for a method you had used before?
  - New prescription – for a method I have **never used before**
  - New prescription – for a method I have **used in the past**
  - Prefer not to answer
- Did the pandemic play a role in the **type** of birth control method you wanted?
  - Yes
  - No
  - Not sure
  - Prefer not to answer
- [*If yes pandemic played role in type*] Please tell us more about how the pandemic played a role in the type of birth control method you wanted: [*textbox*]
- What birth control method(s) were you **prescribed** by [the platform]? Mark all that apply.
  - Birth control pills
  - Patch
  - Ring
  - Emergency contraception
  - None—I was not prescribed any birth control method by [the platform]
  - Prefer not to answer
- *[If was not prescribed any method]* You said were **not prescribed** any birth control method. Please tell us why (select all that apply):
  - I wanted a birth control method not offered at [the platform]
  - I preferred to keep my current birth control method
  - I wanted to learn more about my options before deciding
  - I needed more time to think about my options
  - I don’t know
  - Other (please tell us): [*textbox*]
  - Prefer not to answer
- Did you have to provide any blood pressure information to [the platform]?
  - Yes
  - No
  - I don’t remember
  - Prefer not to answer

*[If had to provide BP info]* How did you get your blood pressure information? [*textbox*]

- *[If prescribed the pill]* What type of **birth control pill** did you receive a **prescription** for? Mark all that apply.
  - Combined pill (contains estrogen and progestin)
  - Progestin-only pill
  - Emergency contraception
  - I don’t know
  - Prefer not to answer
- *[If requested any type of birth control AND was prescribed any birth control method]* Did you get a prescription for the birth control method you originally requested from [the platform]?
  - Yes
  - No
  - Prefer not to answer
- *[If didn’t receive method they had requested]* What were the reason(s) for why you did not get a prescription for the birth control method you originally requested from [the platform]? Please select all that apply.
- A provider told me I had a condition that would make this method risky for me to use
- A provider suggested a different method
- I changed my mind
- I requested the wrong method
- Other (please tell us) *[textbox]*
- Prefer not to answer
- *[If didn’t receive method they had requested]* What did you do for birth control after you did not get a prescription for the method you originally requested from [the platform]? Please select all that apply.
  - Accepted a prescription for a different method from [the platform]
  - Got a method from a different telemedicine service instead
  - Got a method from a different in-person doctor/clinic instead
  - Used a method that did not require a prescription
  - Decided not to use any method
  - None of the above
  - Other (please tell us) *[textbox]*
  - Prefer not to answer
- *[If prescribed any method]* How did you pay for [the platform] services? Please select all that apply.
  - Out-of-pocket - I paid myself
  - Out-of-pocket - Someone else paid for me
  - I used my health insurance
  - Other (please tell us) *[textbox]*
  - I don’t remember
  - Prefer not to answer
- *[If prescribed any method]* Did you **receive** the method you were prescribed from [the platform]?
  - Yes
  - No
  - Prefer not to answer

*[If no]* What were the reason(s) for why you did **not** **receive** the birth control method you were prescribed? Please select all that apply.

- I changed my mind/cancelled my order
- I never received my method in the mail
- I had difficulty picking up my method at the pharmacy
- Other (please tell us) *[textbox]*
- Prefer not to answer

Did you feel you received the information you needed or wanted from [the platform] about your birth control options?

- Yes
- No
- I don’t remember
- I did not need any information from [the platform]
- Prefer not to answer

*[If no*] What information would have been helpful to have when requesting a birth control method from [the platform]? (Select all that apply)

- - Information on side effects
  - Information on effectiveness
  - Information on safety
  - Information on how to use the method
  - Information on other contraceptive methods [the platform] provides
  - Information on methods [the platform] does not provide
  - Other (please tell us) *[textbox]*
  - Prefer not to answer
- *[For current users of contraception that are using a method NOT* from [the platform] *AND not using withdrawal, vasectomy, tubal ligation, or fertility awareness]* You mentioned you are **currently** using [current method], which you received from somewhere other than [the platform]. How was your experience getting a birth control method from [the platform] compared to your experience getting [current method] from this other location/service?
  - Easier from [the platform]
  - Harder from [the platform]
  - The same
  - I don’t know
  - Prefer not to answer
- *[If easier/harder/the same]* Please tell us why.
- *[For current users of contraception who ARE using a method from [the platform]* *AND who reported contraception use in the month before [the platform] care (not withdrawal, vasectomy, tubal ligation, or fertility awareness)]*You mentioned you received [method] **in the month before using [the platform]** from somewhere other than [the platform]. How was your experience getting a birth control method from [the platform] compared to your experience getting [method] from this other location/service?
  - Easier from [the platform]
  - Harder from [the platform]
  - The same
  - I don’t know
  - Prefer not to answer
- *[If easier/harder/the same]* Please tell us why.
- Overall, how satisfied are you with the birth control services you received from [the platform]?
  - Very satisfied
  - Somewhat satisfied
  - Somewhat dissatisfied
  - Very dissatisfied
  - Not sure
  - Prefer not to answer

*[If any other response than ‘Prefer not to answer’]* Please tell us why.

- Would you recommend [the platform] to a friend who needs birth control?
  - Yes
  - No
  - Not sure  *text box*: Please tell us why
  - Depends  *text box*: Please tell us why
  - Prefer not to answer
- What did you like **best** about using [the platform] for birth control? Please select all that apply.
  - It was convenient
  - It was affordable
  - I received high quality of care -> optional text box: Please tell us more about your experience receiving high quality care
  - I liked the video visit
  - I received the method I needed quickly
  - Other (please tell us) *[textbox]*
  - I did not like anything about using [the platform]
  - Prefer not to answer
- What did you like **least** about using [the platform] for birth control? Please select all that apply.
  - It was inconvenient
  - It was expensive
  - I did not feel like I received high quality of care -> optional text box: Please tell us more about why you felt like you did not receive high quality care
  - I did not like having to do a video visit
  - I wanted a video visit but this was not an option
  - It took a long time for me to receive my method
  - Other (please tell us) *[textbox]*
  - There was nothing I disliked about using [the platform] for contraception
  - Prefer not to answer

- How likely are you to use **any telemedicine services** **for birth control** in the future?
  - Very likely
  - Somewhat likely
  - Somewhat unlikely
  - Very unlikely
  - Not sure
  - Prefer not to answer
- *All options (except prefer not to answer) lead to open text box.* Please tell us why.
- How likely are you to use **[the platform] for birth control** in the future?
  - Very likely
  - Somewhat likely
  - Somewhat unlikely
  - Very unlikely
  - Not sure
  - Prefer not to answer
- *All options (except prefer not to answer) lead to open text box.* Please tell us why.

*OTC questions*

*Right now, you need a prescription to get the birth control pill. But it could be possible someday soon for people to get this method “over the counter” without a prescription. With “over-the-counter” access, birth control pills would be available on the shelf at a pharmacy or grocery store just like cough medicine or some allergy pills. You would not need a prescription from a doctor, nurse, or pharmacist. You would not need to talk to anyone about buying birth control pills (not a doctor, pharmacist, or parent) unless you wanted to.*

- Based on the description of this over-the-counter birth control pill, how likely are you to buy and use it?

If you are currently using a long-term method (like the IUD or Implanon/Nexplanon), think about how likely you would be to buy and use an over-the-counter birth control pill the next time you need to change your method.

- - Very likely
  - Somewhat likely
  - Somewhat unlikely
  - Very unlikely
  - Not sure
  - Prefer not to answer
- *All options (except prefer not to answer) lead to open text box* Please tell us why.
- Thank you for completing the survey! Please click SUBMIT to submit your survey and enter the raffle for one of five $100 Amazon gift cards. (You will be directed to a new raffle webpage that is not connected to your survey responses.)
  - SUBMIT

**Raffle Page**

THANK YOU FOR TAKING PART IN THE SURVEY!

To be entered into the raffle for the chance to win one of five $100 Amazon gift cards, please enter your email address (your email address will **NOT** be linked to your survey responses): [email address text field]

Please enter your first name or pseudonym (fake name): [Text box]

Are you interested in being contacted about future studies related to people’s experiences seeking or using birth control pills?

- Yes
- No
