# Supplementary material for: An exploratory study of patients’ experiences with and reasons for using one virtual-only telecontraceptive platform in the United States in 2020–2021
Source: Reprod Health. 2025 Dec 3;22:245. doi: 10.1186/s12978-025-02181-0 (PMC12676766; doi:10.1186/s12978-025-02181-0)
Supplement: Supplementary file 2 — Supplementary Material 2 [file 12978_2025_2181_MOESM2_ESM.docx]

Appendix II Email text sent to potential survey participants

Tell us about your [platform] experience.

Thank you for completing a recent [platform] visit.

At [the platform], we’re always trying to do good in our community to increase healthcare access to all Americans. Through [the platform], we’re helping advance research in telemedicine with the non-profit organization, Ibis Reproductive Health. Ibis is conducting an online survey to better understand your experience using [the platform] during the COVID-19 pandemic. **The information you provide in the survey will be anonymous, which means no identifying information will be collected or shared with [the platform].**

The online survey will take approximately 20 minutes to complete, and you will be entered in a raffle to win one of five $100 Amazon gift cards. You can learn more about the study here [link to study webpage]. If you are interested in participating, you can access the online survey below. We hope you can take part!

Start the survey [link to survey].
