# Supplementary material for: An exploratory study of patients’ experiences with and reasons for using one virtual-only telecontraceptive platform in the United States in 2020–2021
Source: Reprod Health. 2025 Dec 3;22:245. doi: 10.1186/s12978-025-02181-0 (PMC12676766; doi:10.1186/s12978-025-02181-0)
Supplement: Supplementary file 5 — Supplementary Material 5 [file 12978_2025_2181_MOESM5_ESM.docx]

**An exploratory study of patients’ experiences with and reasons for using one virtual-only telecontraceptive platform in the United States in 2020-2021**

Authors: Caila Brander^1^*, Kate Grindlay^1^, Daniel Grossman^2,**^, Carmela Zuniga^1^

^1^Ibis Reproductive Health, Cambridge, MA

^2^ Advancing New Standards in Reproductive Health (ANSIRH), Bixby Center for Global Reproductive Health, Department of Obstetrics, Gynecology and Reproductive Sciences, University of California, San Francisco (UCSF), Oakland, CA, USA

*Co-corresponding author (CB) contact details: 2067 Massachusetts Ave, Suite 320, Cambridge, MA 02140; phone: +1.617.349.0040 ext. 1075; email: cbrander@ibisreproductivehealth.org

**Co-corresponding author (DG) contact details: 1330 Broadway #1100, Oakland, CA 94612; phone: 415-353-4860; email: daniel.grossman@ucsf.edu

Plain English Summary: 161

Abstract word count: 335

Manuscript word count: 4631

Key words:

- Telecontraception
- Telehealth
- Contraception
- COVID-19 pandemic

**Plain English Summary**

A growing number of virtual-only platforms and mobile applications provide contraception services. However, limited research has explored user experiences with these services. In our exploratory study, we conducted an online survey among users obtaining contraception from one specific virtual-only platform for the first time to understand reasons for using telecontraception and what they did or did not like about their experience. We found that most study participants used the platform because of convenience, to save time, and due to lower cost compared to going to a clinic in-person. Many participants had used some form of hormonal birth control before. Most participants were satisfied with their care. However, this virtual platform (like a number of other platforms and mobile applications providing contraception services) did not offer contraception services to people under age 18 and did not accept insurance. Those aged 18-25 were more likely to say they used the service for privacy and to save time and money compared to older participants, which is noteworthy since this group can encounter unique health insurance and financial challenges when trying to get birth control. Even though most people were happy with their care, more can be done to make the service available to all, especially young people, first-time birth control users, and those who would like to use insurance to pay for the platform’s services.

**Abstract**

Background: Telecontraception, whereby one uses a website or mobile application to access contraceptive care, has grown over time, with an increasing number of virtual-only platforms offering this service. There are limited data on user experiences with virtual-only telecontraception, particularly among first-time telecontraception users. The aim of this exploratory study was to understand individuals’ experiences receiving contraceptive services from one virtual-only telecontraceptive platform.

Methods: Individuals aged 18-49 years who had used the platform for contraceptive care for the first time between March 2020-December 2021 were eligible for our online survey. We calculated descriptive statistics, chi-square tests, and Fisher’s exact tests to assess background characteristics, reasons for platform use, methods requested and prescribed, and satisfaction.

Results: Among the 244 participants in our sample, most were first-time telecontraceptive users of any platform (76.2%) and had previously used short-acting hormonal contraception (77.9%). One-third (36.5%) reported COVID-19 played a role in their decision to use the platform. Most used the platform for convenience (81.2%) and to save time (53.7%) or money (43.0%) by not visiting a clinic, and similar proportions reported convenience (81.2%), affordability (55.3%), and the ability to get the method quickly (52.9%) as features they liked best about the platform. Uninsured participants (*versus* insured) were more likely to report using the platform to avoid clinic fees; insured individuals (*versus* uninsured) to save time and avoid inconvenient clinic hours; students (*versus* non-students) and young people (aged 18-24 years *versus* older age groups) for privacy; and Black participants (*versus* other races/ethnicities) for convenience. Most participants were very or somewhat satisfied with the service (86.1%) and would use telecontraception again (82.4%).

Conclusions: Most participants were satisfied with their virtual-only telecontraception experience and reported it was convenient and affordable. However, our exploratory study highlights potential disparities in accessing telecontraceptive services among first-time users of short-acting hormonal contraception, young people aged 18-24, minors, and those without insurance. Future research should explore what access needs telecontraceptive care may be addressing and what more is needed to improve access to contraceptive care in the United States.

**Background**

Telehealth, electronic technologies and services that support at-a-distance healthcare delivery (1), is a promising strategy to enhance access to contraceptive care (2). The use of telehealth for contraceptive care has grown over time, especially since the COVID-19 pandemic when there was a need for alternatives to in-person care (3,4). There are several different models of telehealth that one can use to access contraceptive care in the United States, each with their own set of potential benefits and drawbacks to patients. The number of virtual-only telecontraceptive platforms has increased alongside broader telehealth use in the United States, growing from nine in February of 2018 (5) to 15 by November 2022 (6). Virtual-only telecontraception platforms have different characteristics than telehealth provision of contraception offered by brick-and-mortar facilities that could impact patient experiences and reasons for use. For example, telehealth platforms are unable to offer long-acting reversible contraceptive (LARC) methods, which require an in-person procedure to initiate. Virtual-only platforms do not have facility overhead costs so may offer a lower out-of-pocket consultation fee than clinics (3,6). However, many do not accept insurance overall or for consultation fees specifically, so may cost more for insured individuals (3,5). Health insurance in the United States is still the primary mechanism for individuals to access affordable contraceptive services (7,8). Starting in 2012, the Affordable Care Act mandated that the full cost of contraceptive methods approved by the Food and Drug Administration (FDA) and related services be covered by private health insurance without patient cost sharing (for free) (9). However, a 2024 nationally representative survey found about a quarter of women with private health insurance reported making payments for contraceptive care (10). Additionally, 25.3 million Americans aged under age 65 were uninsured in 2023 for a variety of reasons including that the cost of purchasing insurance was unaffordable, they were not eligible for coverage, they did not need or want health insurance, or because signing up was too difficult (11).

Studies on the use of telehealth for contraceptive care have found high rates of acceptability among users (12–16). While several studies have begun to assess who uses virtual-only telecontraceptive care and why (14,15,17–19), most research to date has focused on contraceptive telehealth care that was delivered by medical practices with in-person components (i.e. not by virtual-only platforms) (12,16,20–22) or did not disaggregate data to distinguish virtual-only platforms specifically (versus practices with in-person components) (13,16). Of existing research on telecontraception provided by virtual-only platforms, only one directly surveyed patients about their experiences and included patients who had previously used the same platform for contraception (15), which may influence reported reasons for using telecontraception and level of satisfaction with the service.

Given the limited data on patients’ first experience receiving contraception from a virtual-only platform, we aimed to conduct an exploratory study to better understand participants’ reasons for choosing and experiences with receiving contraceptive care from a virtual-only platform after the onset of the COVID-19 pandemic.. We also sought to understand how reasons for seeking the platform may have differed in the sample along a variety of demographic characteristics (age, race and ethnicity, census region, income, insurance status, student status, and employment status). Documenting these participant experiences can provide insight into initial expectations about the service, highlight areas for improving patient experiences, and provide useful data for providers and policymakers aiming to understand and improve telecontraceptive experiences

**Methods**

*Study setting*

During the study period (2020-2021), the platform offered oral contraceptives, contraceptive patches, rings, and emergency contraception pills in all 50 states and Washington, DC to people aged 18-50 years. Most visits had a consultation fee that was generally between $15-$40. As with many virtual-only platforms, patients were required to register, provide proof of identity and location, and fill out a set of intake questions. If patients indicated they preferred methods that could not be administered via telehealth, like LARC methods or sterilization, they were told to seek in-person care. After the questionnaire, patients were able to converse in real time with a provider via secure messaging or, when required by state policy or preferred by a patient or provider, via phone or video. Following the consultation, if medically appropriate, patients typically received a 12-month prescription that could be delivered directly to patients’ homes or picked up at a local pharmacy. The platform did not accept health insurance for the cost of the consultation. The cost of the contraceptive method depended on several factors, including the desired product and pharmacy, and this price was not determined or influenced by the virtual platform (6).

*Terminology*

Throughout this paper, we use the term “telecontraception” to refer to the online provision of contraceptive care through a website or smartphone app (17). We specify “virtual-only telecontraception” when these services are provided by virtual companies and platforms that are not connected to in-person practices. We have conceptualized this distinctly from practices that offer contraceptive care both in person and via telehealth.

*Study design*

A research team that was independent from the virtual-only platform conducted an anonymous online survey (Appendix I) with patients aged 18-49 years and who used the platform’s telecontraception service for the first time between March 2020 and December 2021. The online survey, hosted by Qualtrics, included closed-ended questions about participant sociodemographic characteristics and reproductive health history. It also included closed- and open-ended questions on the range of services sought and received, reasons for care-seeking, and acceptability of care received. Participants provided informed consent electronically prior to beginning the survey. We required an answer to each question, which all included a “prefer not to answer” option. Participants could revise their responses before submitting.

*Data collection*

On behalf of the research team, the platform compiled a complete list of 91,123 potentially eligible participants and sent them a single recruitment email with study information and a survey link in July 2022 (Appendix II). The survey was open for four weeks. Upon survey completion, participants could enter a raffle for one of five $100 Amazon gift cards. Contact information for the raffle was collected and stored separately from survey data.

*Dependent variables*

Our primary outcome variables for this study were drawn from a question on reasons for choosing the platform for contraceptives services from a pre-written list, which we asked as follows: “What were your reason(s) for choosing [the platform] for birth control services for the first time? Please select all that apply”. We created new binary variables for each categorical response option: if a participant selected the response option, we coded the associated variable as “yes”, and if they did not select the response option, we coded it as “no”.

Since our study period (2020-2021) occurred during the COVID-19 pandemic, our secondary outcome variable was whether the pandemic played a role in their decision to use virtual-only telecontraception. We created a binary COVID-19 pandemic outcome variable by combining those who selected they had used the platform to avoid an in-person clinic visit during the COVID-19 pandemic and those who responded to a stand-alone question that the COVID-19 pandemic played a role in their decision to use the platform. If they said “yes” to the separate question about whether COVID-19 played a role in their decision to use the platform, they were asked to explain how. All participants were also asked in a stand-alone question if COVID-19 influenced the contraceptive method they requested in their visit, and if they said “yes” they were also asked an open-ended question to explain how. We deductively coded responses to these two open-ended questions in Excel to identify themes. One investigator (CB) completed qualitative coding and analysis, and another (CZ) reviewed results.

*Independent variables*

We grouped contraceptive methods from participants’ contraceptive history into seven categories: barrier (male condoms, female condoms, diaphragm, sponge, spermicide), short-acting hormonal (oral contraception, vaginal ring, shots, patch), long-acting reversible (hormonal or copper intrauterine device, implant), permanent (tubal ligation, partner vasectomy), emergency contraception, withdrawal, and fertility awareness or abstinence methods. We regrouped student status, employment status, and insurance status into binary variables (yes/no/prefer not to answer). We used respondents’ household income, state, and household size to calculate an income variable based on 2020 and 2021 Health and Human Services federal poverty guidelines (≤200% federal poverty guidelines / >200% federal poverty guidelines) (23,24). Participants self-identified their race and ethnicity from a closed-ended response list and a write-in option; respondents that selected multiple race and ethnicity options were re-classified as “more than one race/ethnicity” and one “Pacific Islander” response was grouped with the “Asian” response option to create an “Asian American, Native Hawaiian, and Pacific Islander (AANHPI)” category.

We asked participants whether they provided blood pressure information to the platform; if they said “yes,” we asked how they obtained blood pressure information in an open text box; we coded this open-response text on blood pressure information source into categories, and each response could be included in as many categories as it related to. To assess whether virtual-only telecontraception patients may be interested in additional contraceptive access models outside of a clinical setting, we also asked participants about their theoretical interest in obtaining oral contraceptives over the counter in the future if available, using a 4-point Likert scale (“very likely”, “somewhat likely”, “somewhat unlikely”, “very unlikely”) as well as options for “not sure” and “prefer not answer”; the survey instructed those using a long-term method to think about the next time they needed to change methods.

Throughout the survey, we recoded “other” open-text responses to new or existing categories where appropriate, using footnotes in tables to note when this generated new categories. Missing, don’t know, and prefer not to answer responses are presented in results tables 1-4.

*Data analysis*

After eliminating responses with duplicate IP addresses, locations, or contact information, our analytic sample was determined by including any participants that had answered all outcomes of interest from the survey. We used Stata 15.1 (StataCorp, College Station, TX) to analyze closed-ended questions. We calculated descriptive statistics and used chi-square tests and Fisher’s exact tests to assess relationships between each of our primary outcome variables (the top ten reasons for choosing the platform for contraceptive services) and participant characteristics that we hypothesized *a priori* might predict our outcomes: age, race and ethnicity, census region, income, insurance status, student status, and employment status. We repeated this process to assess relationships between our secondary outcome (whether the pandemic played a role in their decision to use the platform for contraceptive services) and the same participant characteristics. We included race and ethnicity because racial differences have been documented in telecontraceptive use (21,25) and racial health and economic disparities were exacerbated during the COVID-19 pandemic (26,27). Missing, don’t know, and prefer not to answer respones were excluded from chi-square and Fisher’s exact tests.

*Ethics and reporting*

We used the cross-sectional survey STROBE checklist and the online survey CHERRIES checklist in reporting our findings (Appendices III and IV). Allendale Institutional Review Board approved this study (protocol #COVID-19 Telecontraception 2021). Participants provided informed consent via an electronic consent form in order to participate in the study, which provided information about the study’s purpose, procedure, potential risks and benefits, and measures taken to protect confidentiality.

**Results**

Of the 91,123 patients emailed about the survey, 22,500 opened the email and 597 clicked on the survey link. We included 244 responses, excluding those ineligible due to age (n=8), date of first using the platform’s contraceptive services (n=79), or not completing all study outcome questions (n=266). Among those who clicked on the link, we had a 40.9% completion rate (244/597) and an overall response rate of 1.1% (244/22,500).

*Participant characteristics*

Most participants were between 25-34 years old (48.8%) and identified as white (57.0%) (Table 1). About one-third were uninsured at the time of the survey (31.2%) and two in ten had low incomes (20.5%). Roughly one in ten were getting contraception for the first time (11.5%). Many had ever used short-acting hormonal contraceptive methods (77.9%) and used short-acting hormonal methods in the month prior to platform use (56.7%).

| **Table 1: Participant demographic and contraceptive use characteristics, among a sample of first-time users of one virtual-only telecontraception platform during March 2020-December 2021 (N=244)** | |
| --- | --- |
| **Age** | **n (%)** |
| 18-24 | 66 (27.1) |
| 25-34 | 119 (48.8) |
| 35-49 | 59 (24.2) |
| **Race and ethnicity** |  |
| American Indian or Alaska Native | 0 (0) |
| Asian American, Native Hawaiian, and Pacific Islander | 17 (7.0) |
| Black or African American | 25 (10.3) |
| Hispanic, Latina, Latinx, or Spanish origin | 40 (16.4) |
| White | 139 (57.0) |
| More than one race/ethnicity | 16 (6.6) |
| Prefer not to answer | 7 (2.9) |
| **Census** **region (during first use of the platform)** |  |
| West | 50 (20.5) |
| Midwest | 41 (16.8) |
| South | 123 (50.4) |
| Northeast | 29 (11.9) |
| Prefer not to answer | 1 (0.4) |
| **Gender** **identity*** |  |
| Cisgender woman/woman | 237 (97.1) |
| Agender | 2 (0.8) |
| Genderqueer | 1 (0.4) |
| Non-binary | 3 (1.2) |
| Prefer not to answer | 1 (0.4) |
| **Relationship status** |  |
| Never married, single | 74 (30.3) |
| Never married, in a relationship and not living with a partner | 38 (15.6) |
| Never married, in a relationship and living with a partner | 50 (20.5) |
| Married | 54 (22.0) |
| Divorced, widowed, or separated | 25 (10.3) |
| Other | 1 (0.4) |
| Prefer not to answer | 2 (0.8) |
| **Income** |  |
| <200% of the federal poverty guidelines | 50 (20.5) |
| >200% of the federal poverty guidelines | 91 (37.3) |
| Don’t know | 49 (20.1) |
| Prefer not to answer | 54 (22.1) |
| **Insurance status** |  |
| Insured | 156 (63.9) |
| Uninsured | 76 (31.2) |
| Don’t know | 4 (1.6) |
| Prefer not to answer | 8 (3.3) |
| **Student status** |  |
| Yes (part or full time) | 48 (19.7) |
| No | 190 (77.9) |
| Prefer not to answer | 6 (2.5) |
| **Employment status** |  |
| Yes (part or full time) | 182 (74.6) |
| No | 56 (23.0) |
| Prefer not to answer | 6 (2.5) |
| **Contraceptive method(s) ever used before the platform*^†^** |  |
| Barrier methods | 59 (24.2) |
| Short-acting hormonal methods | 190 (77.9) |
| Long-acting reversible | 19 (7.8) |
| Permanent methods | 4 (1.6) |
| Emergency contraception pills | 26 (10.7) |
| Withdrawal | 52 (21.3) |
| Fertility awareness method and abstinence | 6 (2.5) |
| None, first time user | 28 (11.5) |
| **Contraceptive method(s) used in the month prior to using the platform*^†^** |  |
| Barrier methods | 29 (11.9) |
| Short-acting hormonal methods | 138 (56.7) |
| Long-acting reversible | 4 (1.6) |
| Permanent methods | 3 (1.2) |
| Emergency contraception pills | 8 (3.3) |
| Withdrawal | 39 (16.0) |
| Fertility awareness method and abstinence | 0 |
| None, not using method in the month prior | 60 (24.6) |
| * Participants could select multiple responses.  † Categories were based on the following contraceptive options selected in the survey:  Barrier method: male condoms, female condoms, diaphragm, sponge, spermicide  Short-acting hormonal methods: oral contraception, vaginal ring, shots, patch  Long-acting reversible methods: hormonal intrauterine device, copper intrauterine device, implant,  Permanent methods: tubal ligation, partner vasectomy | |

*Prior telehealth and telecontraception experience*

Most participants were first-time telehealth users for any health care need (65.6%) and specifically for contraception (76.2%). Of the 76 who had ever used telehealth before, most had seen their routine provider (54.0%) or used another telehealth company (46.0%). Of the 53 who had used telehealth for contraception before, most had used a virtual-only platform (66.0%) compared to seeing their regular provider (34.0%) or a pharmacist (2.0%) via telehealth (results not shown).

*Reason(s) for platform use*

Most participants reported they decided to use the platform for convenience (81.2%) and the desire to save time (53.7%) or money (43.0%) by not having to visit a clinic. Almost one-third said they used the platform because they did not have insurance (32.0%). Additional top reasons for platform use included inconvenient clinic hours (21.7%), difficulty getting time off or getting childcare (20.1%), not wanting a physical or pelvic exam to get contraception (12.7%), privacy (11.5%), difficulty getting to the clinic (8.2%), and not wanting to use insurance (7.4%). Table 2 shows reasons for platform use (Table 2).

*COVID-19’s impact on platform use*

Over one-third of participants (36.5%) sought the platform’s services due to the pandemic (Table 2). In open-ended follow-up responses, three major themes emerged in the way the pandemic influenced the desire to use the virtual-only platform: 1) wanting to avoid contact with the public while seeking care; 2) difficulty navigating logistical challenges with in-person care such as longer wait times, clinic closures, and reduced hours; and 3) financial concerns due to income or job loss that affected their ability to purchase health insurance or cover the cost of in-person appointments.

| **Table 2: Influences on platform use among a sample of first-time users of one virtual-only telecontraception platform during March 2020-December 2021 (N=244)** | |
| --- | --- |
| **What were your reason(s) for choosing the platform for contraceptive services for the first time?*** | **n (%)** |
| Convenience/easier to get contraception | 198 (81.2) |
| To save time to not have to visit a clinic | 131 (53.7) |
| To save money to not have to pay for a visit to a clinic | 105 (43.0) |
| To avoid in-person clinic visits and/or other COVID-related reasons^§^ | 89 (36.5) |
| I didn’t have insurance | 78 (32.0) |
| Doctor or clinic office hours were not convenient | 53 (21.7) |
| It was hard to get time off from work, school, or to get childcare | 49 (20.1) |
| I didn’t want to get a physical or pelvic exam in order to get contraception | 31 (12.7) |
| Privacy or to get contraception without others knowing | 28 (11.5) |
| It was hard to get to a clinic or pharmacy | 20 (8.2) |
| I didn’t want to use my insurance | 18 (7.4) |
| Some other reason | 7 (2.9) |
| I didn’t have a regular doctor or clinic | 0 |
| *Participants could select multiple responses.  ^§^Incudes respondents that selected this multiple choice option (n=37) as well as those who responded yes versus no, not sure, or prefer not to answer to the following free-standing question: “Did the COVID-19 pandemic play a role at all in choosing to use the Platform for birth control?” (n=81). | |

Chi-square and Fisher’s exact tests revealed differences in some of the top reasons for platform use based on insurance status, age, student status, and race and ethnicity. Those without insurance were more likely than insured participants to choose the platform to avoid paying for clinic visits (57.9% *versus* 37.2%, p=0.003), whereas insured participants were more likely to say clinic hours were inconvenient (25.6% *versus* 13.2%, p=0.03) and that they desired to save time by not having a clinic visit (60.3% *versus* 42.1%, p=0.009). Students were more likely than non-students to report seeking the service for privacy (20.8% *versus* 8.4%, p=0.01), as were people aged 18-24 versus those aged 25-34 and 35-49 years (24.2% *versus* 4.2% and 11.9%, respectively; p<0.001). Young people aged 18-24 were also more likely to report difficulty finding time for an appointment (31.8% *versus* 13.5% and 20.3%; p=0.01) and wanted to save time by not having a clinic visit (66.7% *versus* 49.6% and 47.5%; p=0.045). A greater proportion of Black participants reported seeking the service for convenience (96.0%) compared to AANHPI participants (62.5%), Hispanic/Latinx participants (72.5%), White participants (83.5%), and those reporting more than one race/ethnicity (78.6%) (p=0.04). Income, census region, and employment status were not associated with reasons for platform use. No variables were associated with choosing the platform for contraceptive services due to difficulty getting to a clinic, not having insurance, not wanting to use insurance, not wanting a physical or pelvic exam, or due to the COVID-19 pandemic.

*Contraceptive methods*

Oral contraception was the most common method requested (86.1%) and prescribed (84.0%) (Table 3). Prescriptions for combined oral contraceptive pills were more common than progestin-only pills (63.4% *versus* 15.6%).

*COVID-19 influence on contraceptive method choice*

A few participants (5.7%) reported the pandemic influenced their method preference (Table 3). Open-ended follow-up responses from the participants who reported the pandemic influenced their method choice explained this was due to factors like a desired method being out-of-stock, wanting a more effective method, or wanting a method that did not need to be administered in-person.

| **Table 3: Contraceptive methods requested and prescribed, among a sample of first-time users of one virtual-only telecontraception platform during March 2020-December 2021 (N=244)** | |
| --- | --- |
| **What contraceptive method(s) did you request, if any?*** | **n (%)** |
| Oral contraception | 210 (86.1) |
| Patch | 8 (3.3) |
| Ring | 16 (6.6) |
| Emergency contraception pills | 1 (0.4) |
| Injectable^†^ | 4 (1.6) |
| I did not request a specific method | 5 (2.1) |
| Prefer not to answer | 2 (0.8) |
| Missing | 6 (2.5) |
| **Contraceptive method prescribed*** |  |
| Oral contraception | 205 (84.0) |
| Patch | 8 (3.3) |
| Ring | 16 (6.6) |
| Emergency contraception pills | 4 (1.6) |
| None of the above | 3 (1.2) |
| Prefer not to answer | 8 (3.3) |
| Missing | 6 (2.5) |
| **Type of oral contraception prescribed, if prescribed oral contraception, n=205*** |  |
| Combined oral contraceptive pill | 130 (63.4) |
| Progestin-only pill | 32 (15.6) |
| Don’t know | 38 (18.5) |
| Prefer not to answer | 3 (1.5) |
| Missing | 4 (2.0) |
| **Did the COVID-19 pandemic play a role in the type of contraceptive method you wanted?** |  |
| Yes | 14 (5.7) |
| No | 211 (86.5) |
| Not sure | 12 (4.9) |
| Prefer not to answer | 1 (0.4) |
| Missing | 6 (2.5) |
| * Participants could select multiple responses.  † Response option added from open text coding. | |

*Interest in over-the-counter contraception*

Of the 221 participants who responded to a question about the hypothetical possibility of getting contraceptives over the counter, about three quarters of participants said they would be very (55.2%) or somewhat (17.7%) likely to buy and use an over-the-counter oral contraceptive, while about 8% were very (4.5%) or somewhat (3.2%) unlikely to do so, 10.4% were not sure, and 9.1% preferred not to answer.

*Blood pressure readings*

Overall, 150 participants reported providing blood pressure information to the platform, with many stating that they took a reading at home through an at-home device (42.7%) while others went to a pharmacy or grocery store (26.6%) or provided a reading from a prior health care visit (25.3%); 4% of responses related to blood pressure information source were unclear and thus uncategorizable.

*Participant satisfaction*

Most participants were very (77.5%) or somewhat (8.6%) satisfied with the service they received and would recommend the platform to a friend (84.8%) (Table 4). Additionally, most were very (66.4%) or somewhat (16.0%) likely to use telehealth services for contraception in the future. Participants most liked the convenience (82.8%) and affordability (55.3%) of the service and received their method quickly (52.9%). Some felt the service was expensive (7.4%) or did not provide high quality care (6.2%). In the free-response question about why they felt the service did not provide quality of care, no clear patterns emerged, and responses tended to refer to specific circumstances (i.e. not getting their preferred method due to contraindications, their prescription getting lost in the mail, etc.).

| **Table 4: Satisfaction with contraceptive services, among a sample of first-time users of one virtual-only telecontraception platform during March 2020-December 2021 (N=244)** | |
| --- | --- |
| **Overall, how satisfied are you with the contraceptive services you received from the platform?** | n (%) |
| Very satisfied | 189 (77.5) |
| Somewhat satisfied | 21 (8.6) |
| Somewhat dissatisfied | 8 (3.3) |
| Very dissatisfied | 4 (1.6) |
| Prefer not to answer | 4 (1.6) |
| Missing | 18 (7.4) |
| **Would you recommend the platform to a friend who needs contraception?** |  |
| Yes | 207 (84.8) |
| No | 9 (3.7) |
| Not sure/depends | 6 (2.5) |
| Prefer not to answer | 3 (1.2) |
| Missing | 19 (7.8) |
| **What, if anything, did you like about using the platform for contraception? (Select all that apply)** |  |
| It was convenient | 202 (82.8) |
| It was affordable | 135 (55.3) |
| I received the method I needed quickly | 129 (52.9) |
| I received high quality of care | 40 (16.4) |
| I liked the video visit | 4 (1.6) |
| I did not like anything about using the platform | 3 (1.2) |
| Other | 2 (0.8) |
| Prefer not to answer | 2 (0.8) |
| Missing | 19 (7.8) |
| **What, if anything, did you dislike about using the platform for birth control? (Select all that apply)** |  |
| There was nothing I disliked about using the platform for contraception | 155 (63.5) |
| It was expensive | 18 (7.4) |
| I did not feel like I received high quality of care | 15 (6.2) |
| I did not like having to do a video visit | 13 (5.3) |
| It was inconvenient | 7 (2.9) |
| I wanted a video visit but this was not an option | 6 (2.5) |
| It took a long time for me to receive my method | 4 (1.6) |
| Other | 9 (3.7) |
| Prefer not to answer | 14 (5.7) |
| Missing | 19 (7.8) |
| **How likely are you to use any telemedicine services for contraception in the future?** |  |
| Very likely | 162 (66.4) |
| Somewhat likely | 39 (16.0) |
| Somewhat unlikely | 5 (2.1) |
| Very unlikely | 3 (1.2) |
| Not sure | 10 (4.1) |
| Prefer not to answer | 5 (2.1) |
| Missing | 20 (8.2) |

**Discussion**

This exploratory study complements findings from another virtual-only telecontraception study showing high satisfaction with virtual-only platforms (15), as well as findings that convenience is a valued aspect of telehealth for contraceptive care more broadly (12,13,15,22), including among studies also conducted during the COVID-19 pandemic (12,13,15). Another study on virtual-only telecontraception satisfaction found that the second-leading reason for seeking telecontraceptive care was because virtual-only telecontraceptive users did not have insurance and could not afford a doctor’s visit (15). Similarly, in our study, affordability was a leading reason for seeking virtual-only telecontraceptive care, particularly among uninsured participants, who were more likely than insured participants to choose the platform to avoid clinic visit fees. Our study also identified affordability as a leading reason for satisfaction with the telecontraceptive platform’s care, which other studies conducted during the COVID-19 pandemic did not find (12,13,15).

One-third of participants reported that the COVID-19 pandemic influenced their desire to use the virtual platform for the first time between 2020-2021, and 5% indicated that it influenced their method choice. The COVID-19 pandemic catalyzed federal and state policy shifts in the United States that expanded access to telehealth, such as lifting restrictions on originating site requirements, allowing the use of non-HIPAA compliant platforms under emergency waivers, and expanding Medicaid and Medicare coverage for telehealth services (28). These shifts were felt in contraceptive care, as one provider survey found the proportion of contraception providers in the United States offering telehealth services grew from 11% pre-pandemic to 79% from April 2020-January 2020 (29). A 2021 survey found that 17% of respondents used telehealth for their most recent contraceptive appointment, with about half of those being with virtual-only platforms (13). Future research should be conducted on the post-COVID-19 landscape to better understand the extent to which changes in utilization of telehealth for contraceptive care that emerged during the pandemic period have been sustained and why.

A high proportion of participants in this study reported being uninsured (31%) compared to the general population’s uninsured rate (9%) (11). People may lack insurance for a variety of reasons; a 2023 nationally representative survey found that lack of affordable insurance options, not meeting eligibility requirements, and not feeling health insurance was necessary were the top three reasons adults reported for not having health insurance (11). Though we do not know whether the high proportion of uninsured individuals in our study is representative of the population that uses telecontraception overall, we found that most participants reported that care offered by the platform was acceptable and affordable. One possible reason for this finding could be related to the price of in-person contraceptive care without insurance; while prices vary by practice and fluctuate by region, the national average cost of a visit to an obstetrician-gynecologist without insurance was $280 almost a decade ago in 2016 (30). While prices of telecontraceptive platforms also vary, this study’s platform offered services between $15-40 per consultation and is similar to the prices for other telecontraceptive platforms collated by Ibis Reproductive Health in 2022 (6). These findings could suggest that telecontraceptive platforms are bridging a gap in access to contraception for uninsured individuals, but future research is needed across a variety of platforms to determine if this is true.

Approximately half of adults in the United States have high blood pressure (31), and hypertension is a contraindication for contraceptive methods containing estrogen (32). In this study, most participants reported providing a blood pressure reading to the platform and many reported taking this reading at home. Other research has found utilization of at-home blood pressure monitoring devices may have increased during the pandemic (33). The high proportion of participants who were able to provide a reading might suggest that providing a blood pressure reading was not a barrier for participants. However, it is possible our sample did not capture the experiences of people who may have struggled to provide this blood pressure reading; thus, future research could explore the extent to which providing blood pressure readings is a barrier to using telecontraceptive services and what can be done to alleviate that burden.

Just over a quarter of the sample (27%) were aged 18-24, an age group that face unique barriers to accessing contraceptive care including privacy, finances, and logistics (34–36). We found individuals aged 18-24 were more likely than older age groups to report seeking telecontraceptive care to overcome logistical barriers and due to privacy concerns. Research from the Kaiser Family Foundation found that in 2019, the majority of those aged 18-25 with private insurance were covered as dependents under their parent’s insurance (36). As parents are the primary policyholder, the insurance company may send them the Explanation of Benefits, which describes what health services were used and who received them (36). Members of a health plan can request that sensitive services, such as reproductive health services, not be shared with the policyholder, though whether this request is honored varies by state and insurer (37). Prior research has found that this may motivate some young people to pay out-of-pocket for services covered by insurance, and in some cases avoid accessing health care (35), which may be a significant barrier for some young people. Virtual platforms may be poised to bridge this gap by offering generally lower consultation costs (~$15-40) without the need for insurance, thus better supporting young people’s privacy (6). However, virtual platforms cannot offer the full spectrum of methods a young person might want, including LARC methods, which are highly effective and used by about a quarter of females aged 18-25 (10), but require an in-person insertion procedure. Overall, our findings add to research demonstrating that privacy concerns are forefront for young people seeking contraception (34,38,39) and highlight the need for more states to implement policies requiring insurers to protect the privacy of young adults aged 18-24 receiving reproductive health care services. Overall, the findings from this study support continued research on young people’s use of telecontraception and further expansion of services to this demographic.

Most participants in our study had previously used short-acting hormonal contraception, suggesting the platform may have mostly been meeting the needs of previous contraceptive users. Some reasons for this could be that those using hormonal contraceptives for the first time preferred methods not offered by this platform or encountered barriers that prevent them from using this platform, or it could reflect the fact that the telecontraceptive platform in this study does not provide care to minors. The platform is not unique in this regard; many telecontraceptive platforms only provide to those aged 18 and older (6). Platforms that do offer care to minors can only do so in certain states. As of August 2023, 21 states had restrictions on minors’ ability to consent to contraception without parental involvement, such as only permitting minors who are married or a parent to access contraception without parental involvement (40). The American College of Obstetricians and Gynecologists supports removing state policy restrictions on minors' ability to access contraception without parental notification or consent (41), which could enable virtual-only platforms to expand services to minors in more states.

Our study also found high interest in over-the-counter access to oral contraception among virtual-only telecontraceptive platform users. Overall, 73% reported they would likely use over-the-counter oral contraceptives if available, which is much higher than national levels, in which 39% of adult females of reproductive age report likely use (42,43). The higher level of interest in over-the-counter oral contraception among our study participants highlights that this population, which was already seeking care outside of a clinical setting, may especially benefit from additional expansion to contraceptive access modalities. Our sample included a disproportionately large proportion of uninsured individuals, a background characteristic associated with interest in over-the-counter use in prior research (42,43). After this study concluded, the first over-the-counter oral contraceptive pill was approved by the FDA (44). Yet, the affordability of this method remains a concern as the suggested retail price of $20 is higher than what adults and teens have reported being willing to pay ($15 for adults and $10 for teens) (42).

While the platform in our study was viewed by many participants to be affordable, more can be done to improve affordability of telecontraceptive care including through increased insurance coverage, as most platforms do not currently accept insurance (3,5), as well as expanding that coverage to include virtual-only telecontraception. Under the Affordable Care Act, most private health insurance plans are required to offer contraception with no cost-sharing for beneficiaries; however, a patient’s providers needs to accept their insurance, while many telehealth providers do not accept insurance at all (45). There is evidence that state requirements for parity between telehealth versus in-person reimbursement services can increase access to telehealth for contraception (4), and thus should be considered in states that do not have telehealth reimbursement parity requirements yet. While these options could help make care provided by virtual-only telecontraceptive platforms more accessible, overcoming barriers to contraception will continue to be a multi-pronged approach (46).

*Limitations*

This study has several limitations. Our survey was sent to patients of just one virtual-only platform. The response rate was low and could indicate non-response bias; though this is not uncommon in online surveys using similar methodologies (15), the findings of this study should be viewed as initial exploratory work for future research to follow. Additionally, social desirability bias could have affected responses if participants thought the study was managed by the platform, despite the email stating it was conducted externally. This study was only available in English, which may have limited its ability to reach a diverse population. This study assessed experiences with care after the onset of the COVID-19 pandemic and included periods of time when communities were in lockdowns as well as times where restrictions had eased; thus COVID-related reasons for care-seeking may have fluctuated throughout the study period and may not reflect reasons for care seeking after the acute stages of the pandemic. Nonetheless, household economic wellbeing has declined since the study period and households continue to struggle financially and forgo healthcare needs as a result (47); thus, the main reasons for seeking telecontraceptive services reported in this study, such as saving time and money, are likely to persist.

**Conclusions**

This study found high acceptability of virtual-only telecontraceptive care among first-time users of one virtual-only platform. Participants reported being satisfied due to the convenience, affordability, and time saved using the service. Future research should explore interest in virtual-only telecontraception among a variety of groups that may face greater barriers to accessing contraceptive and telecontraceptive care, including young people, first time short-acting hormonal contraceptive users, and those without insurance. Further research on user characteristics of telecontraception compared to users of other modalities of contraceptive care (such as telehealth services offered by in-person clinics or over-the-counter oral contraception access) could better illuminate what access needs telecontraceptive care may be addressing and what more is needed to improve access to contraceptive care in the United States.

**List of Abbreviations**

AANHPI: Asian American, Native Hawaiian, and Pacific Islander

CHERRIES: The Checklist for Reporting Results of Internet E-Surveys

COVID-19: Coronavirus disease of 2019

FDA: Food & Drug Administration (United States)

HIPAA: Health Insurance Portability and Accountability Act

IP: Internet Protocol (address)

LARC: Long-acting Reversible Contraception

STROBE: Strengthening the Reporting of Observational Studies in Epidemiology

**Declarations**

Ethics approval and consent to participate: Allendale Institutional Review Board approved this study. Participants provided informed consent within the online survey prior to participating in the survey.

Consent for publication: All authors approved the final version of this manuscript for publication.

Availability of data and materials: Due to the confidential nature of the virtual-only telecontraception platform, it is not possible to share the data.

Competing interests: The authors declare no competing interests.

Funding: This study was supported by Arnold Ventures, the Collaborative for Gender + Reproductive Equity, and the David and Lucile Packard Foundation. These funders had no role in the study's design, analysis or interpretation of the data, or the writing of the manuscript.

Authors' contributions: CZ, DG, and KG conceptualized and designed the study. CB and KG supported data collection. CB analyzed the data, with support from CZ, and led interpretation, with support from all authors. CB drafted the manuscript with review and revisions from all authors.

Acknowledgements: The study team would like to thank the people who participated in the study, as well as the team at the virtual-only telecontraception platform for their support identifying and contacting potential participants and for answering the study teams’ questions about service delivery.

**References**

1. American Academy of Family Physicians. Telehealth and Telemedicine [Internet]. 2021 [cited 2023 Jan 27]. Available from: https://www.aafp.org/about/policies/all/telehealth-telemedicine.html

2. Weigel G, Frederiksen B, Ranji U, 2019. Telemedicine in Sexual and Reproductive Health [Internet]. Kaiser Family Foundation. 2019 [cited 2023 Jan 27]. Available from: https://www.kff.org/womens-health-policy/issue-brief/telemedicine-in-sexual-and-reproductive-health/

3. Frederiksen B, Gomez I, 2020. A Look at Online Platforms for Contraceptive and STI Services during the COVID-19 Pandemic [Internet]. KFF. 2020 [cited 2023 Jan 23]. Available from: https://www.kff.org/coronavirus-covid-19/issue-brief/a-look-at-online-platforms-for-contraceptive-and-sti-services-during-the-covid-19-pandemic/

4. Ellison J, Cole MB, Thompson TA. Association of Telehealth Reimbursement Parity With Contraceptive Visits During the COVID-19 Pandemic. JAMA Netw Open. 2022 Apr 11;5(4):e226732.

5. Zuniga C, Grossman D, Harrell S, Blanchard K, Grindlay K. Breaking down barriers to birth control access: An assessment of online platforms prescribing birth control in the USA. J Telemed Telecare. 2020 July;26(6):322–31.

6. Ibis Reproductive Health. Free The Pill. 2022 [cited 2023 Jan 30]. Where Can I Get Birth Control Pills Online? Available from: https://freethepill.org/resources/where-can-i-get-birth-control-pills-online

7. The Regulation of Private Health Insurance [Internet]. KFF. [cited 2025 July 2]. Available from: https://www.kff.org/health-policy-101-the-regulation-of-private-health-insurance/

8. What Is Medicaid? - Medicaid 101 [Internet]. KFF. [cited 2025 July 2]. Available from: https://www.kff.org/health-policy-101-medicaid/?entry=table-of-contents-what-is-medicaid

9. Sobel L, Salganicoff A, Published IG. State and Federal Contraceptive Coverage Requirements: Implications for Women and Employers [Internet]. KFF. 2018 [cited 2025 July 2]. Available from: https://www.kff.org/womens-health-policy/issue-brief/state-and-federal-contraceptive-coverage-requirements-implications-for-women-and-employers/

10. Frederiksen B, Diep K, Published AS. Contraceptive Experiences, Coverage, and Preferences: Findings from the 2024 KFF Women’s Health Survey [Internet]. KFF. 2024 [cited 2025 July 2]. Available from: https://www.kff.org/womens-health-policy/issue-brief/contraceptive-experiences-coverage-and-preferences-findings-from-the-2024-kff-womens-health-survey/

11. Tolbert J, Drake P, Published AD. Key Facts about the Uninsured Population [Internet]. KFF. 2023 [cited 2024 Nov 27]. Available from: https://www.kff.org/uninsured/issue-brief/key-facts-about-the-uninsured-population/

12. Stifani BM, Smith A, Avila K, Boos EW, Ng J, Levi EE, et al. Telemedicine for contraceptive counseling: Patient experiences during the early phase of the COVID-19 pandemic in New York City. Contraception. 2021 Sept 1;104(3):254–61.

13. Lindberg LD, Mueller J, Haas M, Jones RK. Telehealth for Contraceptive Care During the COVID-19 Pandemic: Results of a 2021 National Survey. Am J Public Health. 2022 June;112(S5):S545–54.

14. Nitkowski J. Qualitative analysis of user reviews from Nurx and Planned Parenthood Direct: what user experiences reveal about telecontraception apps. Sex Health. 2022 June 27;19(5):417–26.

15. Frederiksen B, Apr 04 IGP, 2023. Who Uses Telecontraception and Why? A Closer Look at Clients of Four Telecontraception Companies [Internet]. KFF. 2023 [cited 2023 June 2]. Available from: https://www.kff.org/womens-health-policy/issue-brief/who-uses-telecontraception-and-why-a-closer-look-at-clients-of-four-telecontraception-companies/

16. Merz-Herrala AA, Kerns JL, Logan R, Gutierrez S, Marshall C, Diamond-Smith N. Contraceptive care in the United States during the COVID-19 pandemic: A social media survey of contraceptive access, telehealth use and telehealth quality. Contraception. 2023 July;123:110000.

17. Jain T, Schwarz EB, Mehrotra A. A Study of Telecontraception. N Engl J Med. 2019 Sept 26;381(13):1287–8.

18. Martinez KA, Rastogi R, Lipold L, Rothberg MB. Response to requests for contraception in one direct-to-consumer telemedicine service. Contraception. 2020 May 1;101(5):350–2.

19. Jain T, Mehrotra A. Comparison of Direct-to-Consumer Telemedicine Visits With Primary Care Visits. JAMA Netw Open. 2020 Dec 8;3(12):e2028392.

20. Hurtado ACM, Crowley SM, Landry KM, Landry MS. Telehealth contraceptive care in 2018: A quality improvement study of barriers to access and patient satisfaction. Contraception. 2022 Aug 1;112:81–5.

21. Hill BJ, Lock L, Anderson B. Racial and ethnic differences in family planning telehealth use during the onset of the COVID-19 response in Arkansas, Kansas, Missouri, and Oklahoma. Contraception. 2021 Sept 1;104(3):262–4.

22. Shin RJ, Yao M, Akesson C, Blazel M, Mei L, Brant AR. An exploratory study comparing the quality of contraceptive counseling provided via telemedicine versus in-person visits. Contraception. 2022 Aug 1;112:86–92.

23. ASPE [Internet]. [cited 2023 Aug 8]. 2020 Poverty Guidelines. Available from: https://aspe.hhs.gov/topics/poverty-economic-mobility/poverty-guidelines/prior-hhs-poverty-guidelines-federal-register-references/2020-poverty-guidelines

24. ASPE [Internet]. [cited 2023 Aug 8]. 2021 Poverty Guidelines. Available from: https://aspe.hhs.gov/2021-poverty-guidelines

25. Rowley S, Broomfield C, Min J, Quinn S, Campbell K, Wood S. Racial Inequities in Adolescent Contraceptive Care Delivery: A Reproductive Justice Issue. J Pediatr Adolesc Gynecol. 2023 June 1;36(3):298–303.

26. Racial and Ethnic Health Disparities Related to COVID-19 | Health Policy | JAMA | JAMA Network [Internet]. [cited 2023 Nov 6]. Available from: https://jamanetwork.com/journals/jama/fullarticle/2775687

27. Park J. Who is hardest hit by a pandemic? Racial disparities in COVID-19 hardship in the U.S. Int J Urban Sci. 2021 Apr 3;25(2):149–77.

28. Shaver J. The State of Telehealth Before and After the COVID-19 Pandemic. Prim Care. 2022 Dec;49(4):517–30.

29. Comfort AB, Rao L, Goodman S, Raine-Bennett T, Barney A, Mengesha B, et al. Assessing differences in contraceptive provision through telemedicine among reproductive health providers during the COVID-19 pandemic in the United States. Reprod Health. 2022 Apr 22;19:99.

30. Machlin SR, Mitchell EM. Expenses for Office-Based Physician Visits by Specialty and Insurance Type, 2016 [Internet]. Rockville (MD): Agency for Healthcare Research and Quality (US); 2018 [cited 2025 June 27]. Available from: http://www.ncbi.nlm.nih.gov/books/NBK532648/

31. CDC. High Blood Pressure. 2025 [cited 2025 July 14]. High Blood Pressure Facts. Available from: https://www.cdc.gov/high-blood-pressure/data-research/facts-stats/index.html

32. Cameron NA, Blyler CA, Bello NA. Oral Contraceptive Pills and Hypertension: A Review of Current Evidence and Recommendations. Hypertension. 2023 May;80(5):924–35.

33. Home Blood Pressure Monitoring | American Heart Association [Internet]. [cited 2025 July 14]. Available from: https://www.heart.org/en/health-topics/high-blood-pressure/understanding-blood-pressure-readings/monitoring-your-blood-pressure-at-home

34. Brittain AW, Williams JR, Zapata LB, Moskosky SB, Weik TS. Confidentiality in Family Planning Services for Young People: A Systematic Review. Am J Prev Med. 2015 Aug 1;49(2, Supplement 1):S85–92.

35. Campbell-Salome G. “Yes they have the right to know, but…”: Young Adult Women Managing Private Health Information as Dependents. Health Commun. 2019 Aug;34(9):1010–20.

36. Rae M, Claxton G, Published AD. Dependent Coverage for Young Adults in Employer-Sponsored Health Plans [Internet]. KFF. 2024 [cited 2025 June 27]. Available from: https://www.kff.org/private-insurance/issue-brief/dependent-coverage-for-young-adults-in-employer-sponsored-health-plans/

37. Andrews M. States Offer Privacy Protection For Young Adults On Parents’ Health Plan. NPR [Internet]. 2016 June 28 [cited 2025 July 14]; Available from: https://www.npr.org/sections/health-shots/2016/06/28/483836497/states-offer-privacy-protection-for-young-adults-on-parents-health-plan

38. Zuniga C, Wollum A, Katcher T, Grindlay K. Youth Perspectives on Pharmacists’ Provision of Birth Control: Findings From a Focus Group Study. J Adolesc Health Off Publ Soc Adolesc Med. 2019 Oct;65(4):514–9.

39. Sobel L, Beamesderfer A, Published AS. Private Insurance Coverage of Contraception [Internet]. KFF. 2016 [cited 2025 June 27]. Available from: https://www.kff.org/womens-health-policy/issue-brief/private-insurance-coverage-of-contraception/

40. Guttmacher Institute [Internet]. 2016 [cited 2023 Oct 31]. Minors’ Access to Contraceptive Services. Available from: https://www.guttmacher.org/state-policy/explore/minors-access-contraceptive-services

41. Access to Contraception [Internet]. [cited 2023 Nov 8]. Available from: https://www.acog.org/clinical/clinical-guidance/committee-opinion/articles/2015/01/access-to-contraception

42. Grindlay K, Grossman D. Interest in Over-the-Counter Access to a Progestin-Only Pill among Women in the United States. Womens Health Issues Off Publ Jacobs Inst Womens Health. 2018;28(2):144–51.

43. Long M, Frederiksen B, Ranji U, Diep K, Published AS. Interest in Using Over-the-Counter Oral Contraceptive Pills: Findings from the 2022 KFF Women’s Health Survey [Internet]. KFF. 2022 [cited 2024 Nov 27]. Available from: https://www.kff.org/womens-health-policy/issue-brief/interest-using-over-the-counter-oral-contraceptive-pills-findings-2022-kff-womens-health-survey/

44. Belluck P. F.D.A. Approves First U.S. Over-the-Counter Birth Control Pill. The New York Times [Internet]. 2023 July 13 [cited 2023 Aug 8]; Available from: https://www.nytimes.com/2023/07/13/health/otc-birth-control-pill.html

45. Insurance Coverage of Contraceptives | Guttmacher Institute [Internet]. 2016 [cited 2024 Nov 27]. Available from: https://www.guttmacher.org/state-policy/explore/insurance-coverage-contraceptives

46. Salganicoff A, Ranji U. A Focus on Contraception in the Wake of Dobbs. Womens Health Issues. 2023 July 1;33(4):341–4.

47. US Federal Reserve. Economic Well-Being of US Households in 2023 [Internet]. 2024 May [cited 2024 Nov 6]. Available from: https://www.federalreserve.gov/publications/files/2023-report-economic-well-being-us-households-202405.pdf
